# Supplementary material for: Retrospective development of a novel resilience indicator using existing cohort data: The adolescent to adult health resilience instrument
Source: PLoS One. 2020 Dec 10;15(12):e0243564. doi: 10.1371/journal.pone.0243564 (PMC7728188; doi:10.1371/journal.pone.0243564)
Supplement: S1 Table — (DOCX) [file pone.0243564.s001.docx]

| **S1 Table. Characteristics of Candidate Add Health Items Considered for Inclusion in New Instrument** | | | | | |
| --- | --- | --- | --- | --- | --- |
| **Candidate Add Health Item*** | **Variable Name** | **Corresponding CD-RISC Factor(s)**† | **Percent Missing** | **Skewness‡** | **Kurtosis‡** |
| How much do you agree with each statement about you as you generally are now, not as you wish to be in the future? I am not easily bothered by things.* | H4PE14 | 1, 2 | 0.23 | 0.379 | 2.849 |
| How often do you feel isolated from others?* | H4MH2 | 3 | 0.05 | 0.653 | 3.407 |
| In the last 30 days, how often have you felt confident in your ability to handle your personal problems?* | H4MH4 | 1 | 0.05 | -1.012 | 4.205 |
| In the last 30 days, how often have you felt that difficulties were piling up so high that you could not overcome them?* | H4MH6 | 1 | 0.10 | 0.772 | 3.924 |
| In the last 30 days, how often have you felt that you were unable to control the important things in your life?* | H4MH3 | 4 | 0.10 | 0.645 | 3.472 |
| How much do you agree with each statement about you as you generally are now, not as you wish to be in the future? I hardly ever expect things to go my way.* | H4PE15 | 1,4 | 0.24 | -0.413 | 3.411 |
| How much do you agree with each statement about you as you generally are now, not as you wish to be in the future? I'm always optimistic about my future.* | H4PE7 | 3 | 0.22 | 0.953 | 5.243 |
| How much do you agree with each statement about you as you generally are now, not as you wish to be in the future? I go out of my way to avoid having to deal with problems in my life.* | H4PE33 | 1,2 | 0.20 | -0.517 | 3.090 |
| How much do you agree or disagree with the following statement? There is little I can do to change the important things in my life.* | H4PE37 | 4 | 0.27 | -0.823 | 5.540 |
| How much do you agree or disagree with the following statement? Other people determine most of what I can and cannot do.* | H4PE38 | 4 | 0.20 | -0.991 | 5.371 |
| How much do you agree or disagree with the following statement? There are many things that interfere with what I want to do.* | H4PE39 | 4 | 0.22 | -0.277 | 2.664 |
| How much do you agree or disagree with the following statement? There is really no way I can solve the problems I have.* | H4PE41 | 1, 2, 3 | 0.20 | -0.834 | 6.446 |
| How much do you agree with each statement about you as you generally are now, not as you wish to be in the future? Overall, I expect more good things to happen to me than bad.* | H4PE23 | 3,4 | 0.20 | 1.283 | 6.737 |
| How committed are you to your relationship with {first name}? | H4RD10 | 3 | 0.28 | - | - |
| Were you and {fill initials} living together at that time? | H4PG10 | 3 | 0.41 | - | - |
| What is your present religion? | H4RE1 | 5 | 0.33 | - | - |
| How often do you turn to your religious or spiritual beliefs for help when you have personal problems, or problems at school or work? | H4RE11 | 5 | 0.23 | - | - |
| How important (if at all) is your religious faith to you? | H4RE9 | 5 | 0.28 | - | - |
| How many close friends do you have? (Close friends include people whom you feel at ease with, can talk to about private matters, and can call on for help.) | H4WS4 | 3 | 1.27 | - | - |
| Overall, how often (do/did) you have the freedom to make important decisions about what you (do/did) at work and how you (do/did) it? | H4LM23 | 4 | 0.13 | - | - |
| How much do you agree with each statement about you as you generally are now, not as you wish to be in the future? I get stressed out easily. | H4PE22 | 2 | 0.29 | - | - |

*These items were ultimately included in the AHRI.

†**CD RISC** **Factor 1**: Personal competence, high standards and tenacity; **Factor 2**: Trust in one's instincts, tolerance of negative affect, strengthening effects of stress; **Factor 3**: Positive acceptance of change and secure relationships; **Factor 4**: Control; **Factor 5**: Spiritual Influences

‡ Only reported for items included in factor analysis
