# Supplementary figures and images for: Retrospective development of a novel resilience indicator using existing cohort data: The adolescent to adult health resilience instrument
Source: PLoS One. 2020 Dec 10;15(12):e0243564. doi: 10.1371/journal.pone.0243564 (PMC7728188; doi:10.1371/journal.pone.0243564)

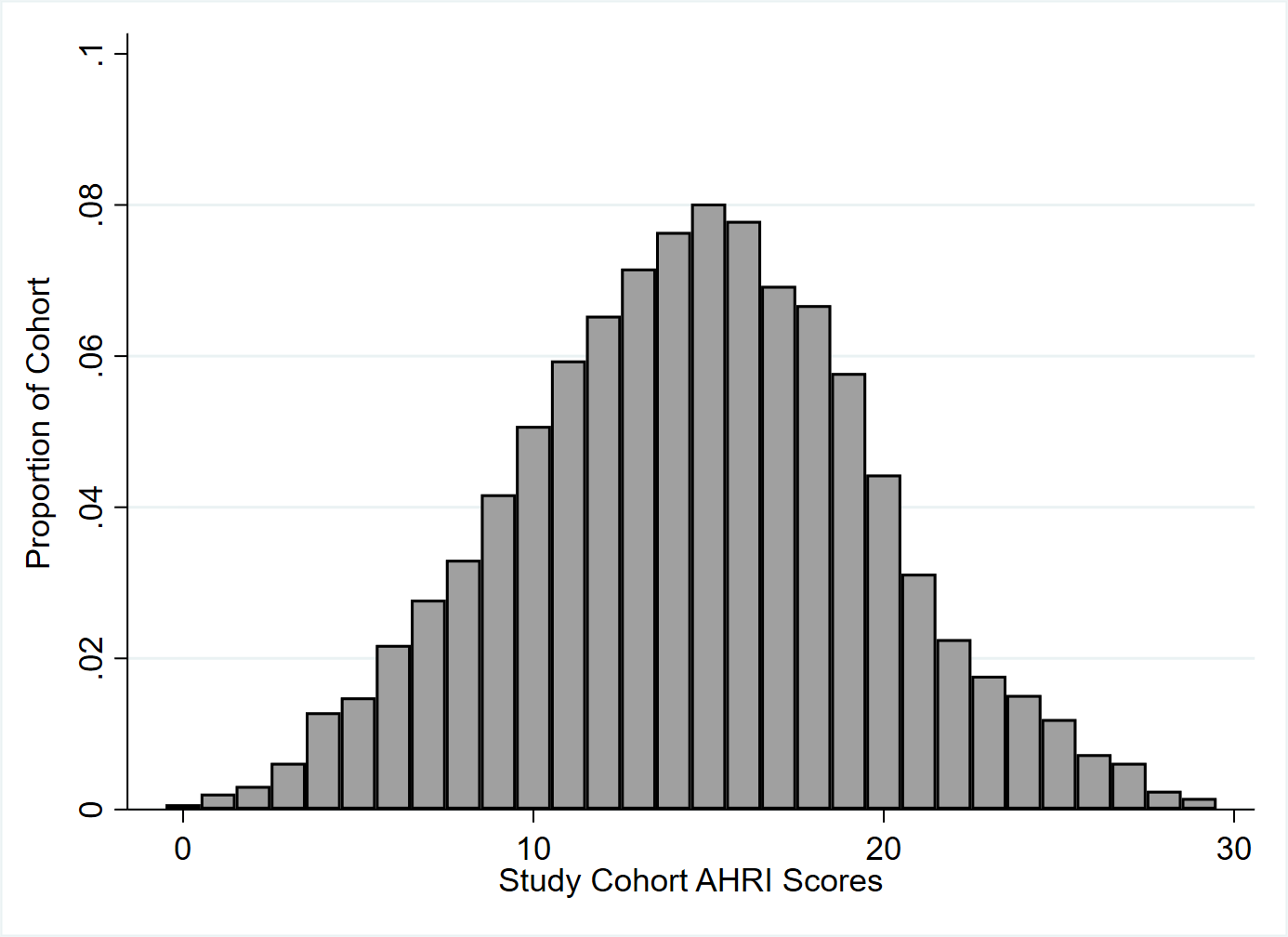

Supplement: S1 Fig — (TIF) [file pone.0243564.s002.tif]

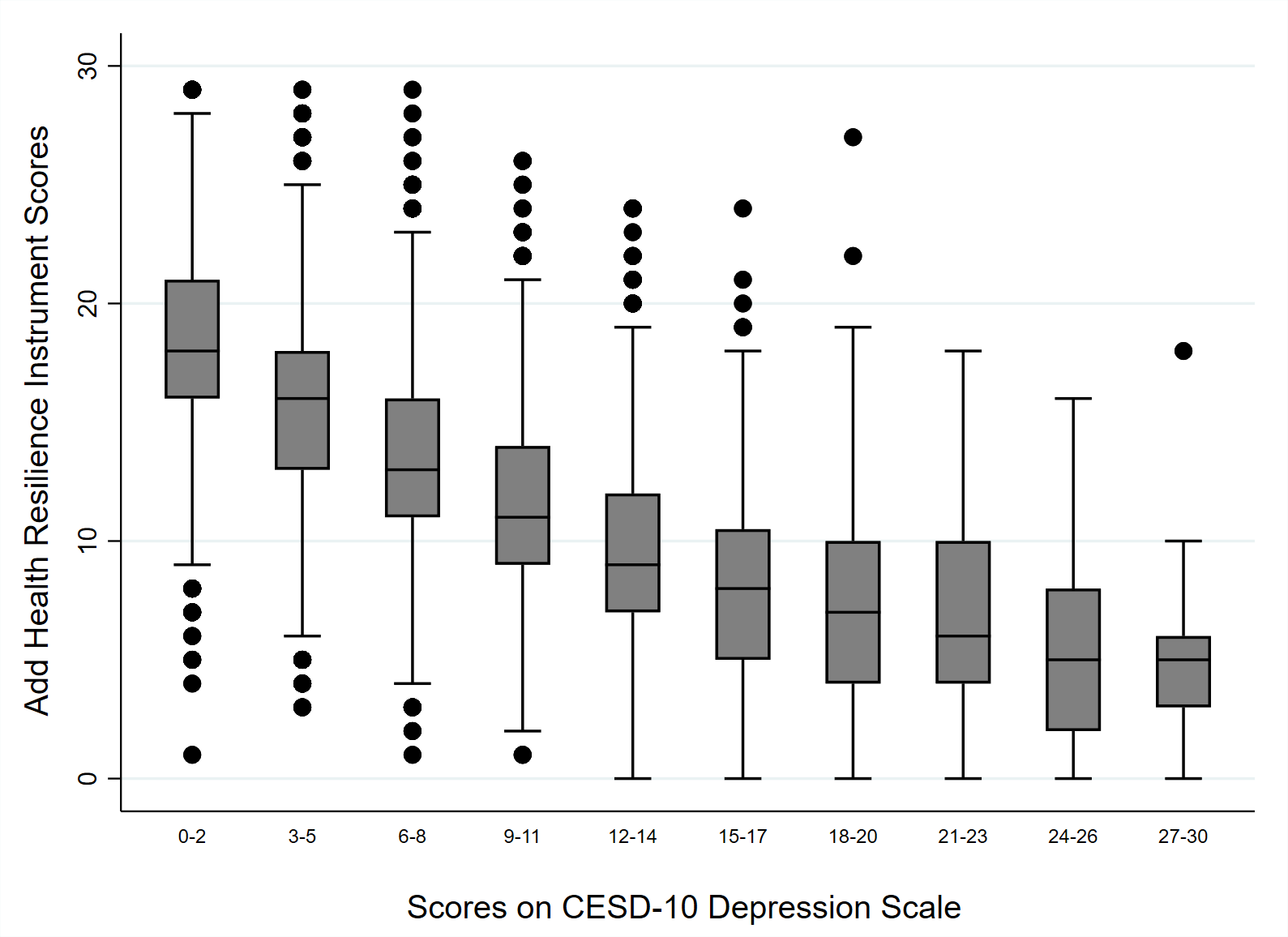

Supplement: S2 Fig — (TIF) [file pone.0243564.s003.tif]
