# Supplementary material for: Retrospective development of a novel resilience indicator using existing cohort data: The adolescent to adult health resilience instrument
Source: PLoS One. 2020 Dec 10;15(12):e0243564. doi: 10.1371/journal.pone.0243564 (PMC7728188; doi:10.1371/journal.pone.0243564)
Supplement: Attachment — Submitted filename: PONE-D-20-14860_comments to the author.pdf [file pone.0243564.s004.pdf]

1. Is the manuscript technically sound, and do the data support the conclusions?

The proposed AHRI can be a useful tool for researchers working with the Add Health study. The manuscript is reasonably sound, and the evidence seems to support the conclusions the authors present. However, some technical matters require more attention and discussion.

I base the following six observations and recommendations on my comparison of the manuscript content with desiderata for factor analysis listed in page 94 of Bandalos's and Finney's 2010 "Factor Analysis: Exploratory and Confirmatory," in Hancock's and Mueller's (editors) *The Reviewer's Guide to Quantitative Methods in the Social Sciences,* published by Routledge.

1. The authors introduce the concept of resilience only very briefly. Although they mention typically used components of the latent construct, a fuller discussion of the theory behind or conceptual understanding of this construct would be helpful. For example, in the Discussion section, the authors mention that there is an ongoing debate about the best way to understand resilience: as a presumably fixed personality trait, as a behavioral outcome, or as a dynamic process. It stands to reason that the use of the concept may vary depending on different research questions, so the authors should make clear those research questions and the conceptual understanding or theory that links the concept of resilience to upstream predecessors or downstream outcomes. Making the conceptual understanding of resilience explicit will also help evaluate how well the candidate and selected Add Health items operationalize the concept.

2. Authors should indicate whether their factor analysis is *exploratory* or *confirmatory* (EFA, or CFA) and justify their decision, as each type of analysis serves a different purpose. The general guidance is to use the first type for newly developed constructs or when the theoretical basis is weak and to use CFA when the structure of the variable is well-established, as when using an independent sample to test a well-studied structure.

3. The authors should also indicate whether any of the variables selected as candidates for the factor analysis are not continuous. If so, they should note whether the variables are dichotomous or scales with less than five points. Such items may result in biased solutions and require a different type of analysis. Authors should identify all items, and including the variables' codes will be helpful to researchers who wish to adopt the new instrument presented in this research paper. Also, the authors could have discussed whether the candidate items provide good-enough coverage of the dimensions of the latent concept. A table of the CD-RISC's items, organized by domain, with the corresponding Add Health items lined up would be beneficial and illustrate whether any domains from the CD-RISC cannot be operationalized.

4. A discussion of the size of the analytical sample is missing: over 35 percent of the Wave IV sample was lost. Did one or a few items result in this loss of observations? If so, how does this affect the operationalization of the latent concept? Were non-responses concentrated among certain age, gender, racial or ethnic groups? If so, what impact does that have on the applicability of the proposed AHRI?

5. Difficulty factors that lead to misleading factor solutions may arise when variables with similar skewness and kurtosis can result in artefactual factors. To rule this out, or identify such factors, the authors should provide a summary of the candidate item's descriptive statistics (mean, standard deviation, and values of skewness and kurtosis). Perhaps the table I proposed above can include CD-RISC items first, the corresponding candidate ASRI items next, then the percent missing responses for each item, and the descriptive statistics last.

6. I expected some discussion of the dimensionality of the chosen solution. The authors indicated that the CD-RISC operationalizes five domains, but the AHRI only has three. For example, the authors excluded the religiosity items from the selected solution, so the proposed AHRI does not operationalize the domain of *spiritual influences.* Is this not an important limitation of the proposed AHRI? Can the religiosity items from Add Health be retained to provide coverage for that domain, balancing a fuller operationalization with a weaker mathematical solution? What is the other domain not covered in the chosen solution, and can alternative solutions solve this?

The following seven observations concern my reading of the *Methodology* and *Results* sections:

7. I am not familiar with the evaluation of convergent validity. Based on its description (line 154 on), it seems to consist in evaluating the proposed AHRI through its correlation with a validated scale for depression, the CESD-10, which is empirically associated with extant resilience scales. I would appreciate a brief description of justification for this type of validation. Am I correct in thinking it consists of logically inverting the independent variable (resilience, which is theoretically upstream from the outcome of depression) with the dependent variable?

8. The second factor in the chosen factor solution, "Social Support / Feeling Overwhelmed," is problematic. Having good social support and feeling overwhelmed strike me as related by not necessarily covarying concepts. A person who perceives the support of friends and family may feel overwhelmed by particularly stressful situations or traumatic events, while a person without support may feel overwhelmed more frequently.

9. I appreciated the analysis of average resilience scores for different demographic groups on page 11. However, given that some of the participants in the Add Health study may not have completed college, become financially independent, or had time to establish themselves in their careers *yet*, the comparisons by educational attainment and household income may not be the most meaningful.

10. Finally, I may be misinterpreting information on Tables 4 and 5 (on pages 11 and 12), but it seems that the confidence intervals for the scores of some of the comparison groups overlap. For example, at the top of Table 4, the mean AHRI score for Female is 13.8 with SD of 5.0, so the 95% confidence interval would run from around 3.8 to 23.8, roughly speaking, and would overlap the mean for Male, which is 14.4. Therefore, the means for Female and Male are not significantly different at the 95% confidence level, let alone at the 99.9% level.

2. Has the statistical analysis been performed appropriately and rigorously?

Yes, it has. However, as indicated above, some of the decisions the authors made require more discussion.

3. Have the authors made all data underlying the findings in their manuscript fully available?

No, the authors are contractually barred from sharing the restricted-use data from the Add Health study. However, Add Health makes a probability sample from the same wave the authors used available for public use. Confirming the results of the author's analysis should be possible with that data.

4. Is the manuscript presented in an intelligible fashion and written in standard English?

Yes, but the authors should proofread for punctuation errors. They can also tighten the structure of the narrative to better guide the reader through their work.

Specific suggestions:

1. Some necessary commas are missing. For example, as written in line 116, the last domain of the CD-RISC seems to be, "control and spiritual influences," when these are two distinct domains. I recommend the use of the Oxford comma, which adds clarity in lists. The authors may consider revising their manuscript to avoid using the passive voice, although this may be a field-specific preference.
2. I recommend the authors use the full name of the Add Health study in the first mention: National Longitudinal Study of Adolescent to Adult Health. The authors may be more familiar with cohort studies and retrospective analyses, but I consider it is more accurate to describe Add Health as a longitudinal study and to describe the author's work as a cross-sectional analysis. It seems to be that a retrospective analysis would require predicting outcomes in Wave 4 (the wave from which the authors drew data) using independent variables from one of the earlier waves, but that is not the case. It would also be preferable if the authors distinguished the terms *study* and *dataset* (Add Health is a study, and several datasets are available for the various questionnaires that make up the study.) **It is important to note that Add Health participants should not be described as patients, as being a patient was not a selection for inclusion.**
3. Wave IV, conducted in 2008, is not the Add Health study's original wave of data collection (line 155).
4. The description of the resilience scale developed by Hitlin and Elder previously belongs in the *Introduction,* together with other extant evidence is presented, rather than appearing towards the end of the paper.
